# Characteristics of pulmonary artery catheter use in multicenter ICUs in Japan and the association with mortality: a multicenter cohort study using the Japanese Intensive care PAtient Database

**DOI:** 10.1186/s13054-023-04702-4

**Published:** 2023-10-28

**Authors:** Kentaro Fukano, Yusuke Iizuka, Seiya Nishiyama, Koichi Yoshinaga, Shigehiko Uchino, Yusuke Sasabuchi, Masamitsu Sanui

**Affiliations:** 1 Department of Anesthesiology and Critical Care Medicine, Jichi Medical University Saitama Medical Center, 1-847 Amanuma-tyo, Omiya-ku,, Saitama-shi, Saitama-ken 330-8503 Japan; 2 Department of Real-World Evidence, Graduate School of Medicine, The University of Tokyo, 7-3-1 Hongo, Bunkyo-ku, Tokyo, 113-8654 Japan; 3 Division of Critical Care, Department of Anesthesiology and Critical Care Medicine, Jichi Medical University Hospital, 3311-1, Yakushiji, Shimotsuke, Tochigi 329-0498 Japan

**Keywords:** Pulmonary artery catheter, Swan–Ganz catheter, Intensive care unit, Cardiogenic shock, Open-heart surgery

## Abstract

**Background:**

It has been 50 years since the pulmonary artery catheter was introduced, but the actual use of pulmonary artery catheters in recent years is unknown. Some randomized controlled trials have reported no causality with mortality, but some observational studies have been published showing an association with mortality for patients with cardiogenic shock, and the association with a pulmonary artery catheter and mortality is unknown. The aim of this study was to investigate the utilization of pulmonary artery catheters (PACs) in the intensive care unit (ICU) and to examine their association with mortality, taking into account differences between hospitals.

**Methods:**

This is a retrospective analysis using the Japanese Intensive care PAtient Database, a multicenter, prospective, observational registry in Japanese ICUs. We included patients aged 16 years or older who were admitted to the ICU for reasons other than procedures. We excluded patients who were discharged within 24 h or had missing values. We compared the prognosis of patients with and without PAC. The primary outcome was hospital mortality. We performed propensity score analysis to adjust for baseline characteristics and hospital characteristics.

**Results:**

Among 184,705 patients in this registry from April 2015 to December 2020, 59,922 patients were included in the analysis. Most patients (94.0%) with a PAC in place had cardiovascular disease. There was a wide variation in the frequency of PAC use between hospitals, from 0 to 60.3% (median 14.4%, interquartile range 2.2–28.6%). Hospital mortality was not significantly different between the PAC use group and the non-PAC use group in patients after adjustment for propensity score analysis (3.9% vs 4.3%; difference, − 0.4%; 95% CI − 1.1 to 0.3; *p* = 0.32). Among patients with cardiac disease, those with post-open-heart surgery and those in shock, hospital mortality was also not significantly different between the two groups (3.4% vs 3.7%, *p* = 0.45, 1.7% vs 1.7%, *p* = 0.93, 4.8% vs 4.9%, *p* = 0.87).

**Conclusions:**

The frequency of PAC use varied among hospitals. PAC use for ICU patients was not associated with lower hospital mortality after adjusting for differences between hospitals.

**Supplementary Information:**

The online version contains supplementary material available at 10.1186/s13054-023-04702-4.

## Introduction

It has been over 50 years since the pulmonary artery catheter (PAC) was first commercially introduced and reported by Swan and Ganz in 1970 [1–5]. In 1996, a secondary analysis of a prospective observational study using propensity score matching indicated that the use of PAC was associated with increased mortality rates [6]. This was followed by a series of randomized controlled trials (RCTs) conducted in the 2000s, none of which demonstrated a prognostic benefit from PAC [7–12]. Subsequently, it was reported that the utilization of PACs decreased by 67.8% from 1999 to 2013 [13].

However, PACs have not been completely phased out. There have been a rising number of reports on PAC usage in patients suffering from heart failure and cardiogenic shock [14–20]. In the USA, the use of PACs among patients undergoing cardiac surgery showed an upward trend from 2010 to 2014 [21]. However, the specific conditions under which PAC was used in the intensive care unit (ICU) after 2015 were unknown. There were several RCTs to evaluate causality between PAC use and mortality in the ICU between 2002 and 2006. The ESCAPE study included patients with severe symptomatic heart failure [10]. Patients except for cardiovascular patients were included in the other RCTs [7–9, 11, 12]. There were no RCTs conducted about the causality between PAC use and mortality in patients with post-open-heart surgery or cardiogenic shock. Therefore, it is unclear whether those RCTs demonstrate a causal relationship between PAC use and mortality in patients in whom we currently use PAC. In fact, several observational studies have been conducted after those RCTs to evaluate the association of PAC use with prognosis. Those studies have suggested that the use of PAC is associated with lower mortality rates in patients with cardiogenic shock [22]. The association between PAC use and decreased mortality in ICU settings is also not well known.

Therefore, our objectives in this study are to characterize the current indications of PAC use in various diseases and to examine the hospital characteristics, patients' demographic and clinical characteristics, vital signs, laboratory data, and outcomes, such as hospital mortality and ICU mortality. We also aim to identify the patient demographics in which PAC usage is potentially beneficial.

## Methods

### Study design and setting

The present study represents a retrospective analysis of the Japanese Intensive Care PAtient Database (JIPAD) [23]. Managed by the Japanese Society of Intensive Care Medicine, JIPAD is a multicenter prospective observational registry of critically ill patients admitted to Japanese ICUs. As of 2021, 82 hospitals were participating in this registry. JIPAD commenced data collection in 2014 and began requiring the input of catecholamine variables, such as the use of dopamine, norepinephrine, dobutamine, and epinephrine, in April 2018. This study received approval from the Institutional Review Board at Jichi Medical University Saitama Medical Center as well as the administration office of JIPAD (S22-106). Due to the anonymous nature of the data, the requirement for informed consent was waived.

### Participant selection

In April 2018, JIPAD began requiring the input of catecholamine variables. As catecholamines were deemed essential for this study, we utilized data from April 2018 to December 2020. The study included patients aged 16 years or older who were admitted to the ICU for reasons other than procedural interventions (e.g., central vein catheter placement). Patients were excluded from the study if they were discharged within 24 h or if their data contained missing values.

### Data collection and measurements

All data were prospectively collected from JIPAD. The details of this data collection process have been previously reported [23]. The following data were used for this study: age, sex, weight, height, diagnosis text, diagnosis code (“Diagnosis text” is a more detailed classification than diagnosis code. For example, the diagnosis code “other cardiac surgery” includes both open-heart surgery and non-open-heart surgery.), the root of ICU admission (1. transfer from the ward, 2. transfer from the emergency room, 3. ICU admission following elective surgery, 4. admission after emergency surgery, 5. other reasons), ICU readmission, comorbidities (chronic heart failure, chronic respiratory failure, use of immunosuppressants, undergoing hemodialysis), acute physiologic assessment and chronic health evaluation (APACHE III score), use of dopamine/dobutamine/epinephrine/norepinephrine during the first 24 h of ICU admission, PAC use during the first 24 h after ICU admission, high flow nasal cannula use, noninvasive positive pressure ventilation use, date/time of initiation and discontinuation of mechanical ventilation, intra-aortic balloon pump (IABP) use, venoarterial extracorporeal membrane oxygenation (VA ECMO) use, venovenous extracorporeal membrane oxygenation (VV ECMO) use, intermittent renal replacement therapy (IRRT) use, continuous renal replacement therapy (CRRT) use, ICU discharge outcome, hospital discharge outcome, date of hospital admission/discharge, date/time of ICU admission/discharge, vital signs and laboratory values during the first 24 h after ICU admission, facility identification number, type of hospital (1. university hospital, 2. public hospital, 3. private hospital), the number of hospital beds, the number of ICU beds, the number of ICU doctors, the number of ICU nurses, the number of ICU clinical engineers, and the number of ICU pharmacists.

Patients were classified according to the diagnosis texts and diagnosis codes, and these classifications were used to create a new variable named “reason for ICU admission.” Hospitals were divided into three groups (low frequency, medium frequency, and high frequency) based on the frequency of PAC use.

### Measurements

The primary outcome of this study was hospital mortality. Secondary outcomes included hospital length of stay, ICU mortality, ICU length of stay, use of high flow nasal cannula, noninvasive positive pressure ventilation, mechanical ventilation (including duration), IABP, VA ECMO, VV ECMO, IRRT, and CRRT.

### Statistical analysis

Continuous variables were represented as the means (standard deviation; SD) or medians (interquartile range; IQR), while categorical variables were depicted as frequencies and percentages. Student's t test or the Wilcoxon rank-sum test was used to compare continuous variables, depending on their distribution. Categorical variables were compared using the chi-square test where appropriate; otherwise, Fisher’s exact test was employed.

To account for differences in baseline characteristics between patients with and without PAC, we implemented a propensity score method. We considered potential confounders that were feasibly linked to both PAC selection and outcome. These included age, sex (with female as a reference), reason for ICU admission (with thoracic aortic aneurysm and acute aortic dissection as references), the route of ICU admission, comorbidities, maximum lactate level at baseline, APACHE III score, use of dopamine, norepinephrine, dobutamine, and epinephrine, along with all vital signs and laboratory values within the first 24 h post-ICU admission. A logistic regression model with the generalized estimating equation was used to calculate propensity scores for PAC use, considering the abovementioned independent variables. An absolute standardized difference of less than 10% was interpreted as evidence of balance.

Patients who used PAC were matched to those who did not use a 1:1 nearest-neighbor matching algorithm, with a caliper of 20% of the standard deviation of the propensity scores on the logit scale. We conducted before and after propensity score matching analyses for the overall group, the group of patients with cardiac disease, the group of patients after open-heart surgery, and the group of patients with cardiac disease with shock or mechanical circulatory device. We used generalized estimating equations fitted with logistic regression models in the matched groups to assess the association between PAC and mortality adjusting for clustering within hospitals. Open-heart surgery was defined as cardiac surgery with an open chest. Cardiac disease was defined as open-heart surgery plus cardiogenic shock, cardiac arrest, aortic aneurysm, aortic dissection, congestive heart failure, and acute myocardial infarction. Cardiac disease with shock or mechanical circulatory support was defined as cardiac disease with catecholamine and/or mechanical circulatory support such as IABP, VA ECMO, and VV ECMO.

R software (version 4.2.2) was employed for analysis and graphing. All P values were two-tailed, with *p* < 0.05 deemed statistically significant. (After the manuscript was written by the lead author and reviewed and revised by the coauthors, English language proofreading was conducted using ChatGPT®.)

## Results

**Fig. 1:**
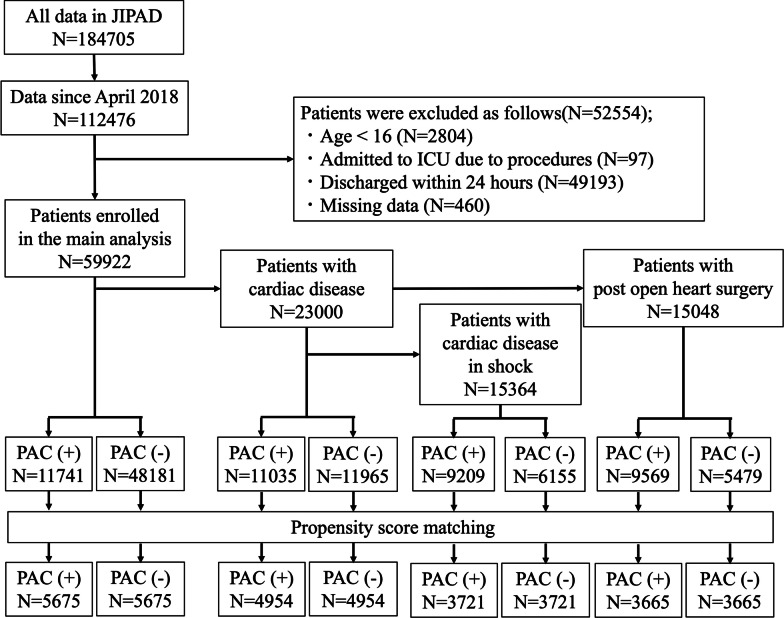
Patient flow in unmatched patients

From April 2015 to December 2020, 184,705 patients were registered, with 112,476 patients from April 2018 to December 2020 being included in this study. Patients aged 15 years old or younger, those admitted for procedures, those discharged within 24 h, and those with missing data were excluded from the analysis (Fig. 1). This left 59,922 patients for consideration in this study. Among these, 23,000 patients (38.4%) had cardiac disease, 15,048 patients (25.1%) were post-open-heart surgery, and 15,364 patients (25.6%) had cardiac disease with shock.

**Fig. 2:**
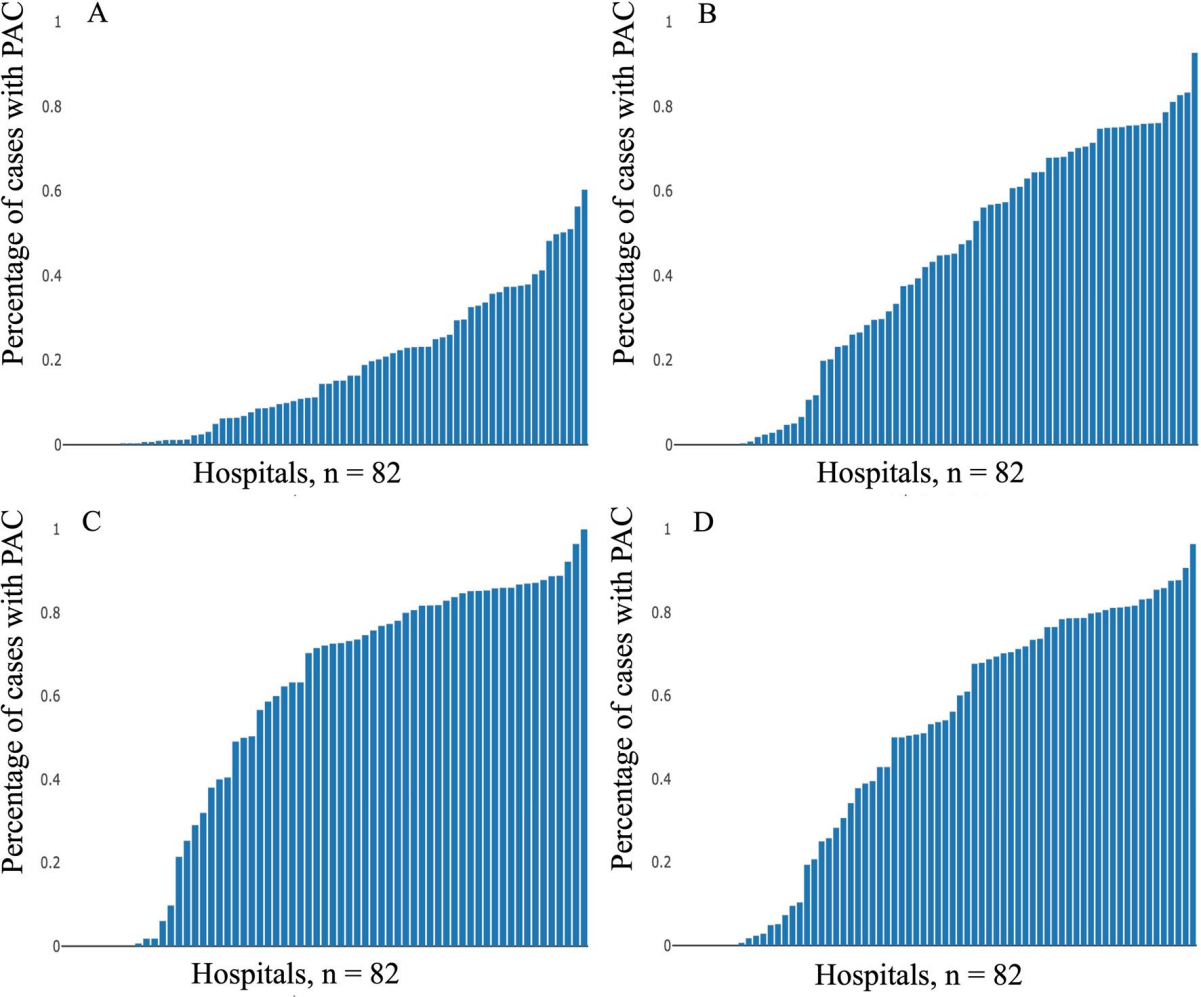
Ratios of patients with pulmonary artery catheter in unmatched all patients (**A**), patients with cardiac disease (**B**), post-open-heart surgery (**C**) and cardiac disease with shock and device (**D**). PAC, pulmonary artery catheter

The distribution of patients with PAC in each hospital is illustrated in Fig. 2. PAC use varied substantially among hospitals, with some facilities employing PACs for up to 60% of all ICU patients, while others did not use them at all. The distribution of patients with PAC also differed among patient groups. The characteristics of hospitals sorted by PAC use frequency are summarized in Additional file 1: Table S1. It was noted that university hospitals and facilities with more hospital and ICU beds were more likely to use PAC.

The baseline characteristics of the study patients are summarized in Table 1. Of the total patients, 11,741 (19.6%) received PAC, while 48,181 (80.4%) did not. PACs were used more frequently in university hospitals than in public and private hospitals (64.5% vs 22.6% vs 13.0%). Most patients (94.0%) with a PAC in place had cardiovascular disease, and 51.0% of such patients were managed with PAC. Among them, valve surgery patients were most common (37.7%), followed by coronary artery bypass grafting (CABG) (21.2%), thoracic aortic aneurysm (TAA) and acute aortic dissection (AAD) patients (17.4%). PAC was infrequently used for other medical (2.6%) or surgical diseases (3.4%). A very small number of patients with acute respiratory distress syndrome (ARDS) (0.5%) or septic shock (1.5%) used PAC. Among patients who used PAC, 72.8% were post-elective surgery. Patients with PAC had higher rates of a history of heart failure or receiving dialysis than those without PAC. Patients without PAC had higher rates of a history of immunosuppression than those without PAC. There were significant differences in APACHE III scores between the groups of patients with and without PAC. The use of catecholamines showed significant differences between the two groups. Additionally, excluding max creatinine, vital signs and laboratory values during the first 24 h post-ICU admission differed between the groups (Additional file 1: Table S2). Primary and secondary outcomes are detailed in Table 2. All outcomes, except for VV ECMO use, showed significant differences between the two groups.

**Table 1:** Patient characteristics in all unmatched and matched patients

	Unmatched			Matched		
	PAC(−)	PAC(+)	SMD	PAC(−)	PAC(+)	SMD
Number of patients	48,181	11,741		5675	5675	
Age, median year, IQR	71 [59, 79]	71 [63, 77]	0.06	71 [62, 78]	71 [62, 78]	< 0.01
Male (%)	30,674 (63.7)	7703 (65.6)	0.04	3671 (64.7)	3694 (65.1)	0.01
Height, median cm, IQR	162 [154, 168]	162 [154, 169]	0.05	162 [154, 168]	162 [154, 169]	0.03
Body weight, median kg, IQR	58.0 [49.5, 67.6]	60.0 [51.7, 68.9]	0.11	60.0 [51.2, 70.0]	60.0 [51.7, 69.0]	0.01
BMI, median kg/m^2^, IQR	22.4 [19.8, 25.2]	22.9 [20.6, 25.6]	0.1	23.0 [20.5, 25.7]	22.9 [20.5, 25.5]	0.04
Hospital type (%)			0.35			0.04
University hospital	22,776 (47.3)	7569 (64.5)		3012 (53.1)	3088 (54.4)	
Public hospital	15,817 (32.8)	2649 (22.6)		1760 (31.0)	1752 (30.9)	
Private hospital	9588 (19.9)	1523 (13.0)		903 (15.9)	835 (14.7)	
Reason for ICU admission (%)			2.33			0.07
Open-heart surgery						
TAA/AAD	1781 (3.7)	1920 (16.4)		1143 (20.1)	1043 (18.4)	
Valve surgery	1509 (3.1)	4165 (35.5)		1304 (23.0)	1335 (23.5)	
CABG	972 (2.0)	2336 (19.9)		857 (15.1)	878 (15.5)	
Valve + CABG	185 (0.4)	617 (5.3)		158 (2.8)	165 (2.9)	
Other open-heart surgery	1032 (2.1)	531 (4.5)		359 (6.3)	339 (6.0)	
Other cardiac surgery	340 (0.7)	213 (1.8)		159 (2.8)	144 (2.5)	
Cardiovascular disease						
Cardiogenic shock	165 (0.3)	144 (1.2)		86 (1.5)	92 (1.6)	
Cardiac arrest	1627 (3.4)	275 (2.3)		263 (4.6)	243 (4.3)	
AMI	1585 (3.3)	359 (3.1)		320 (5.6)	317 (5.6)	
Aortic aneurysm	1119 (2.3)	144 (1.2)		103 (1.8)	137 (2.4)	
Congestive heart failure	1650 (3.4)	331 (2.8)		286 (5.0)	288 (5.1)	
Other medical disease	16,604 (34.5)	311 (2.6)		275 (4.8)	302 (5.3)	
Other surgical disease	19,612 (40.7)	395 (3.4)		362 (6.4)	392 (6.9)	
Root of ICU admission (%)			0.9			0.03
Transfer from ward	2276 (4.7)	208 (1.8)		155 (2.7)	163 (2.9)	
Transfer from ER	11,987 (24.9)	963 (8.2)		819 (14.4)	866 (15.3)	
After elective surgery	15,862 (32.9)	8543 (72.8)		3319 (58.5)	3333 (58.7)	
After emergency surgery	7697 (16.0)	1230 (10.5)		754 (13.3)	712 (12.5)	
Other	10,359 (21.5)	797 (6.8)		628 (11.1)	601 (10.6)	
ICU readmission (%)	3522 (7.3)	315 (2.7)	0.21	205 (3.6)	208 (3.7)	< 0.01
Comorbidities (%)						
Heart failure	669 (1.4)	641 (5.5)	0.23	224 (3.9)	230 (4.1)	0.01
Respiratory failure	893 (1.9)	130 (1.1)	0.06	89 (1.6)	79 (1.4)	0.01
Immunosuppression	3773 (7.8)	282 (2.4)	0.25	204 (3.6)	198 (3.5)	0.01
Hemodialysis	2893 (6.0)	995 (8.5)	0.1	414 (7.3)	415 (7.3)	< 0.01
APACHE III median, IQR	65 [49, 85]	61 [50, 73]	0.22	62 [49, 77]	61 [50, 76]	0.02
Catecholamine use (%)						
Dopamine	4082 (8.5)	2979 (25.4)	0.46	1240 (21.9)	1210 (21.3)	0.01
Noradrenaline	15,247 (31.6)	5477 (46.6)	0.31	2157 (38.0)	2210 (38.9)	0.02
Dobutamine	5059 (10.5)	7249 (61.7)	1.26	2609 (46.0)	2605 (45.9)	< 0.01
Adrenaline	750 (1.6)	371 (3.2)	0.11	140 (2.5)	141 (2.5)	< 0.01

*PAC* Pulmonary artery catheter, *SMD* Standardized mean difference, *IQR* Interquartile range, *TAA* Thoracic aortic aneurysm, *AAD* Acute aortic dissection, *CABG* Coronary artery bypass grafting, *AMI* Acute myocardial infarction, *BMI* Body mass index, *ICU* Intensive care unit, *ER* Emergency room, *APACHE* Acute physiologic assessment and chronic health evaluation, Catecholamine use within 24 h after ICU admission

**Table 2:** Interventions and outcome in all unmatched and matched patients

	Unmatched			Matched		
	PAC(−)	PAC(+)	*p* value	PAC(−)	PAC(+)	*p* value
Number of patients	48,181	11,741		5675	5675	
HFNC (%)	5592 (11.6)	2325 (19.8)	< 0.01	1035 (18.2)	1112 (19.6)	0.07
NPPV (%)	4240 (8.8)	1871 (15.9)	< 0.01	709 (12.5)	887 (15.6)	< 0.01
Mechanical ventilation (%)	24,894 (51.7)	10,895 (92.8)	< 0.01	4294 (75.7)	4953 (87.3)	< 0.01
Duration, median hours, IQR	44 [16, 123]	17 [10, 44]	< 0.01	18 [10, 61]	17 [9, 63]	0.06
VV ECMO (%)	269 (0.6)	64 (0.5)	0.92	32 (0.6)	44 (0.8)	0.21
IABP (%)	1150 (2.4)	1331 (11.3)	< 0.01	455 (8.0)	786 (13.9)	< 0.01
VA ECMO (%)	460 (1.0)	530 (4.5)	< 0.01	167 (2.9)	353 (6.2)	< 0.01
IRRT (%)	3525 (7.3)	980 (8.3)	< 0.01	419 (7.4)	426 (7.5)	0.83
CRRT (%)	4786 (9.9)	1384 (11.8)	< 0.01	604 (10.6)	685 (12.1)	0.02
ICU mortality (%)	6608 (13.7)	676 (5.8)	< 0.01	522 (9.2)	434 (7.6)	< 0.01
Hospital mortality (%)	2417 (5.0)	357 (3.0)	< 0.01	243 (4.3)	221 (3.9)	0.32
ICU length of stay, median days, IQR	26 [15, 49]	26 [18, 41]	< 0.01	22 [16, 37]	25 [17, 41]	< 0.01
Hospital length of stay, median days, IQR	3 [1, 6]	3 [2, 5]	< 0.01	3 [2, 5]	3 [2, 6]	< 0.01

*PAC* Pulmonary artery catheter, *IQR* Interquartile range, *HFNC* High flow nasal cannula, *NPPV* Noninvasive positive pressure ventilation, *IABP* Intra-aortic balloon pump, *VA ECMO* Venoarterial extracorporeal membrane oxygenation, *VV ECMO* Venovenous extracorporeal membrane oxygenation, *IRRT* Intermittent renal replacement therapy, *CRRT* Continuous renal replacement therapy, *ICU* Intensive care unit

The patient characteristics following propensity score matching are presented in Table 1. All variables were well balanced between the two groups, with a standard mean difference of less than 0.1. Vital signs and blood test results were also similar (Additional file 1: Table S3). The primary and secondary outcomes are presented in Table 2. Hospital mortality was not significantly different between the two groups (3.9% vs 4.3%, *p* = 0.32).

**Fig. 3:**
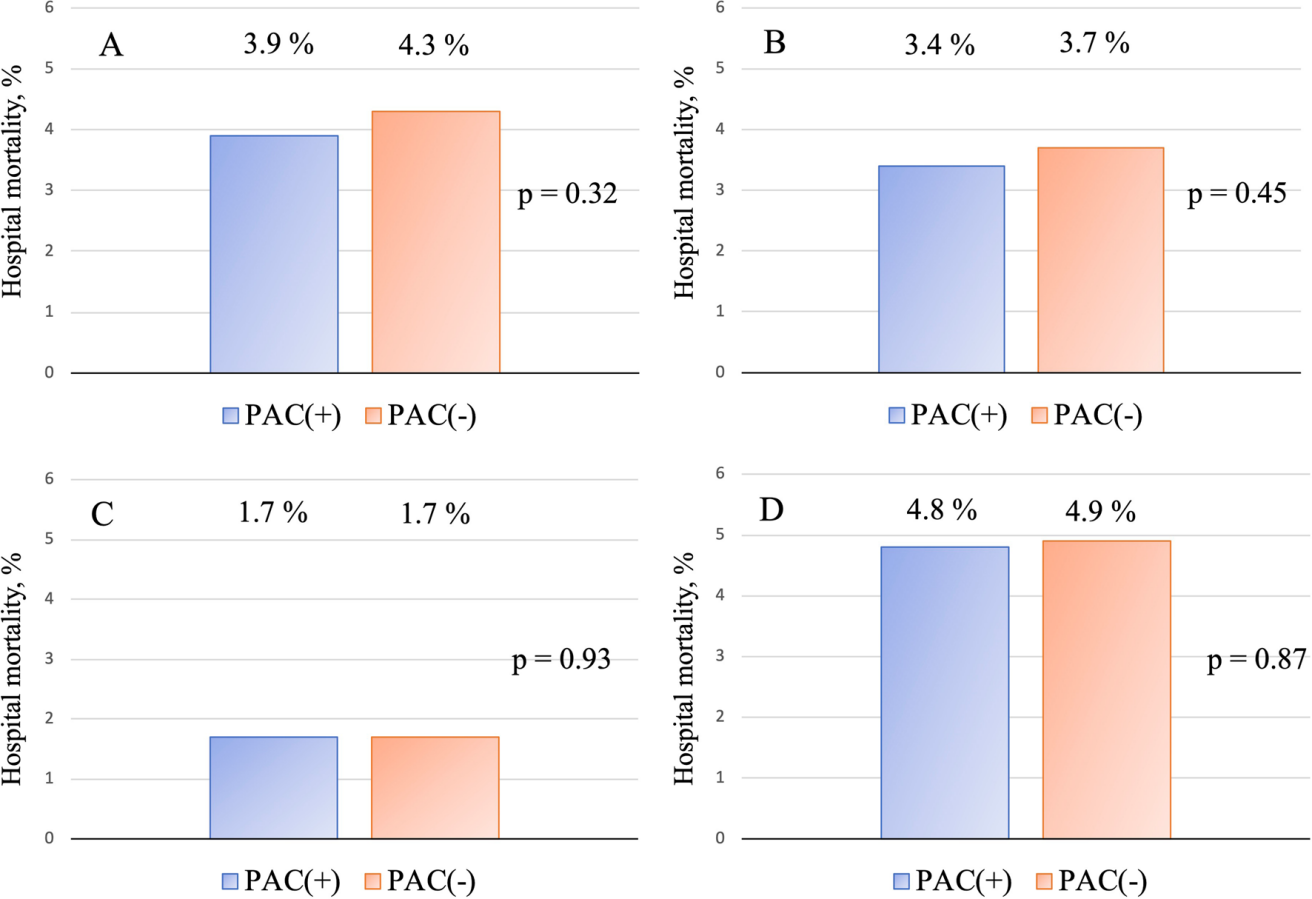
Hospital mortality in matched all patients (**A**), patients with cardiac disease (**B**), post-open-heart surgery (**C**) and cardiac disease with shock and device (**D**). PAC, pulmonary artery catheter

After propensity score matching, patient characteristics within the subgroups of patients with cardiac disease, post-open-heart surgery, and cardiac disease in shock are summarized in Additional file 1: Tables S4, S6, and S8. All variables were well balanced between the two groups within these subpopulations. The primary outcomes are shown in Fig. 3, and the other primary and secondary outcomes are shown in Additional file 1: Tables S5, S7, S9. Among patients with cardiac disease, those with post-open-heart surgery and those in shock, hospital mortality was not significantly different between the two groups (3.4% vs 3.7%, *p* = 0.45, 1.7% vs 1.7%, *p* = 0.93, 4.8% vs 4.9%, *p* = 0.87, respectively) (Fig. 3).

## Discussion

We analyzed PAC utilization in the ICU using a multicenter registry. PAC continued to be widely used, with 19.6% of all patients who stayed in the ICU for more than 24 h utilizing it. Most patients (94.0%) with a PAC in place had cardiovascular disease, and 51.0% of such patients were managed with PAC. Specifically, PAC was frequently used in patients post-open-heart surgery (63.6%) and in those with cardiogenic shock (46.6%). It was also more commonly used at university hospitals and hospitals with a large number of hospital beds and ICU beds. However, the frequency of PAC use varied considerably among hospitals, ranging from 0 to 60.3%. To account for the differences between hospitals, we performed propensity matching analysis and found that hospital mortality was not significantly different between patients with and without PAC. Subgroup analyses revealed that hospital mortality was also similar in patients with cardiovascular disease, patients post-open-heart surgery and cardiogenic shock.

Our study found that PACs continue to be utilized in ICUs, predominantly for patients with cardiovascular disease. Despite the findings of the RCTs in the 2000s that reported no benefit of PAC usage on patient prognosis [7–12], more recent studies have reported sustained use of PACs in patients with heart failure and cardiogenic shock [14–20]. For example, a report from Japan using an acute heart failure registry found that PAC was used in 16.8% of heart failure patients presenting with hypotension or catecholamine use between 2007 and 2011 [24]. Our study showed PAC usage in 16.7% of heart failure patients admitted to the ICU, suggesting that PAC use in severe heart failure patients has not changed since the publication of the RCTs. A systematic review of observational studies evaluating PAC effectiveness in cardiogenic shock patients also reported that PAC was used in 33% of these patients [22], with PAC use ranging from 9.6 to 81.6% in various studies [17, 26]. The frequency of PAC use in our study was 46.6% for cardiogenic shock patients, consistent with previous studies. Another study on PAC usage in ICU patients excluding post-cardiovascular surgery cases reported a decrease in PAC use from 10.8% between 2001 and 2003 to 6.2% from 2004 to 2008 [25]. Our study found that only 4.8% of patients, excluding postcardiac surgery patients, were managed with PAC. This decrease in PAC use in non-cardiovascular disease coupled with steady use in cardiovascular disease has resulted in PACs being almost exclusively used in patients with cardiovascular disease, especially postcardiac surgery. The need for more proactive hemodynamic assessment by PAC in patients with cardiovascular disease may be due to several factors, including the rising number of noncardiac comorbidities such as dialysis and pulmonary hypertension due to an aging population and the challenges in assessing hemodynamic status due to advanced therapies such as mechanical circulatory support (MCS) [14, 17]. Indeed, our study observed a more frequent use of dialysis and MCS in patients managed with PAC.

PAC usage was found to vary from hospital to hospital in our study, being more frequent in university hospitals. It has been reported that university hospitals tend to utilize PAC more often [25], and our study further revealed a positive correlation between the number of hospital beds and PAC usage. The frequency of PAC usage in patients undergoing CABG and valve surgeries with or without CABG has also been reported to vary widely among hospitals [18]. A single-center report from 2022 investigating the frequency of PAC usage in patients with cardiac disease found that PAC was used in 22.9% of such patients [27], which is much lower than in our study (48.0%). We also observed a wide disparity in the frequency of PAC usage among hospitals for all ICU patients, patients with cardiac disease, post-open-heart surgery patients, and patients with cardiac disease in shock (Fig. 2). Intensivists have been found to interpret PAC results differently [28]. Given that PAC usage could impact prognosis, further research and training in PAC usage may be necessary [29].

Although it was concluded in the 2000s that PAC did not reduce mortality [7–12, 30, 31], the demographics of patients currently with a PAC in place differ from those in the RCTs, in which patients commonly suffered from sepsis and ARDS. In our study, a very small number of patients with ARDS (0.5%) or septic shock (1.5%) used PAC, and 94.0% of study patients with a PAC in place had cardiovascular disease. Some observational studies have suggested that PAC is not associated with reduced mortality in patients undergoing postoperative cardiovascular surgery, who are presently the most commonly treated with PAC, but PAC is associated with reduced mortality in patients with cardiogenic shock [14–17, 22, 24, 26, 27, 32]. However, these previous observational studies did not account for disparities between hospitals in their analysis, although the frequency of PAC usage (and possibly its interpretation) varies significantly among hospitals. Thus, we performed propensity analysis adjusting for differences between hospitals. Our analysis confirmed some of the previous results that the use of PAC was not associated with better prognosis (e.g., post-cardiac surgery) but contradicted the others (e.g., cardiogenic shock). New RCTs may need to be conducted to investigate the causality between PAC use and mortality reduction in patients with cardiovascular disease, especially cardiogenic shock.

Previous studies have reported that the frequency of PAC usage in patients undergoing cardiovascular surgery ranges from 25.8 to 39% [19, 21, 32]. In our study, the usage frequency was higher than that reported in these studies, and the use of PAC was not associated with better prognosis. This finding is consistent with a previous report, which found that PAC use may have limited benefit in cardiac surgery [32]. PAC might not help and might even generate intervention in conditions, albeit critical, with very low mortality, such as open-heart surgery patients without cardiogenic shock. However, there are reports that PAC was associated with an improved prognosis in cardiac surgery patients who developed cardiogenic shock, suggesting that it may be useful in patients who develop cardiogenic shock or in those who receive mechanical circulatory support after cardiac surgery [33, 34]. Our study did not analyze cardiogenic shock following open-heart surgery, since catecholamines were frequently administered and lactate levels often rose in these patients. Future studies are needed to ascertain the optimal indications for PAC in postcardiac surgery patients.

### Strengths and limitations

To the best of our knowledge, this study showed the most recent trend of PAC in ICUs since 2015 and this is the first study to report the frequency of PAC use by each hospital and the first study to analyze hospital mortality after adjusting for clustering between hospitals. However, this study also has several limitations. First, since this study is based on secondary use of the national registry, there is a possibility of information bias, although JIPAD collects data to maintain accuracy and minimize bias [23]. Second, the association between PAC use and prognosis may be influenced by selection bias. To mitigate this potential bias, we performed a propensity score matching analysis and adjusted for confounders. Third, because this is an observational study, only known confounders were adjusted. However, unlike previous studies, our analysis included patient background, hospital characteristics, vital signs, laboratory data, and differences in the frequency of PAC use between hospitals, suggesting that we adjusted for as many confounders as possible. Unfortunately, we were only able to capture data on PAC use within first 24 h of ICU admissions and some interventions may have been performed after we started to use PAC. The association of PAC use with reduced mortality and some interventions may have been influenced. Fourth, given that the study was conducted in Japan, there is a question of generalizability, although similar trends in PAC use reported in previous studies. For example, the interpretation of how frequently PACs in our study may be distorted　due to the vastly different mortality and different care patterns in patients with post-open-heart surgery from in other ICU patients (i.e., short duration of stay in ICUs and low mortality). Fifth, this study did not examine the devices used in patients who did not receive PAC. However, no devices have been reported to be compatible with the parameters measured by PAC [35, 36]. Finally, we think how PAC was used is more important than whether PAC is used or not since PAC is only a monitor and not a treatment. It was unclear in our study that the practice of using PAC among centers (knowledge of the method, indications, competence) could influence the results because we had no data about how PAC was used. However, we assumed that PAC utilization practices were consistent across hospitals and a logistic regression model with a generalized estimating equation was used to calculate propensity scores for PAC use.

## Conclusions

PACs continue to be used in the management of critically ill patients, particularly those with cardiac disease, although the frequency of PAC use varies from hospital to hospital. The use of PAC in the ICU for patients was not associated with lower hospital mortality after adjusting for differences between hospitals. Considering the continuing use of PAC in patients with cardiac disease, further studies are needed to evaluate the potential benefits (or harms) of PAC in these patient groups.

### Supplementary Information

**Additional file 1**. **Table S1**: Hospital characteristics in the groups classified by the frequency of PAC use. **Table S2**: Vital signs and blood tests in all unmatched patients. **Table S3**: Vital signs and blood tests in all matched patients. **Table S4**: Patients characteristics in unmatched and matched patients with cardiac disease. **Table S5**: Interventions and outcome in unmatched and matched patients with cardiac disease. **Table S6**: Patients characteristics in unmatched and matched patients with post-open-heart surgery. **Table S7**: Interventions and outcome in unmatched and matched patients with post-open-heart surgery. **Table S8**: Patients characteristics in unmatched and matched patients with cardiac disease with shock and/or device. **Table S9**: Interventions and outcome in unmatched and matched patients with cardiac disease with shock and/or device.

## Data Availability

The data that support the findings of this study are available from JIPAD, but restrictions apply to the availability of these data, which were used under license for the current study and are not publicly available. Data are, however, available from the authors upon reasonable request and with permission of JIPAD.

## Abbreviations

PAC: Pulmonary artery catheter
RCT: Randomized controlled trial
ICU: Intensive care unit
ARDS: Acute respiratory distress syndrome
JIPAD: The Japanese Intensive care PAtient Database
APACHE: Acute physiologic assessment and chronic health evaluation
IABP: Intra-aortic balloon pump
VA ECMO: Venoarterial extracorporeal membrane oxygenation
VV ECMO: Venovenous extracorporeal membrane oxygenation
IRRT: Intermittent renal replacement therapy
CRRT: Continuous renal replacement therapy
SD: Standard deviation
IQR: Interquartile range
CABG: Coronary artery bypass grafting
TAA: Thoracic aortic aneurysm
AAD: Acute aortic dissection
ARDS: Acute respiratory distress syndrome
MCS: Mechanical circulatory support
