# Supplementary material for: Characteristics of pulmonary artery catheter use in multicenter ICUs in Japan and the association with mortality: a multicenter cohort study using the Japanese Intensive care PAtient Database
Source: Crit Care. 2023 Oct 28;27:412. doi: 10.1186/s13054-023-04702-4 (PMC10612322; doi:10.1186/s13054-023-04702-4)
Supplement: Supplementary file 1 — Additional file 1. Table S1: Hospital characteristics in the groups classified by the frequency of PAC use. Table S2: Vital signs and blood tests in all unmatched patients. Table S3: Vital signs and blood tests in all matched patients. Table S4: Patients characteristics in unmatched and matched patients with cardiac disease. Table S5: Interventions and outcome in unmatched and matched patients with cardiac disease. Table S6: Patients characteristics in unmatched and matched patients with post-open-heart surgery. Table S7: Interventions and outcome in unmatched and matched patients with post-open-heart surgery. Table S8: Patients characteristics in unmatched and matched patients with cardiac disease with shock and/or device. Table S9: Interventions and outcome in unmatched and matched patients with cardiac disease with shock and/or device. [file 13054_2023_4702_MOESM1_ESM.docx]

Supplementary Table 1 Hospital characteristics in the groups classified by the frequency of PAC use

|  | Low frequency | Medium frequency | High frequency | P value |
| --- | --- | --- | --- | --- |
| Number of hospitals | 25 | 24 | 25 |  |
| PAC use median %, IQR | 1 [0, 2] | 14 [10, 16] | 36 [29, 40] | <0.001 |
| Hospital type (%) |  |  |  | <0.001 |
| University hospital | 3016 (20.0) | 12038 (56.6) | 15291 (64.9) |  |
| Public hospital | 7373 (48.8) | 6117 (28.8) | 4976 (21.1) |  |
| Private hospital | 4707 (31.2) | 3116 (14.6) | 3288 (14.0) |  |
| Number of Hospital beds, median, IQR | 550 [500, 992] | 664 [603, 731] | 722 [613, 1086] | <0.001 |
| Number of ICU beds, median, IQR | 10 [8, 14] | 12 [10, 14] | 14 [12, 18] | <0.001 |
| Number of ICU doctors , median, IQR | 2 [2, 3] | 2 [1, 3] | 2 [1, 5] | <0.001 |
| Number of ICU nurses, median, IQR | 34 [29, 35] | 38 [29, 53] | 42 [30, 49] | <0.001 |
| Number of ICU CEs, median, IQR | 1 [1, 2] | 1 [0, 1] | 1 [1, 4] | <0.001 |
| Number of ICU pharmacists, median, IQR | 1 [1, 2] | 1 [1, 2] | 1 [1, 2] | <0.001 |

PAC: Pulmonary artery catheter, IQR: Interquartile range, ICU: Intensive care unit, CE: Clinical engineer, Small group includes hospitals: The small group includes hospitals with lower frequency of PAC use. Large group: The large group includes hospitals with higher frequency of PAC use. Medium group: Between small and large group.

Supplementary Table 2 Vital signs and blood tests in all unmatched patients

|  | PAC(-) | PAC(+) | SMD |
| --- | --- | --- | --- |
| Number of patients | 48181 | 11741 |  |
| Vital signs |  |  |  |
| Heart rate-max, bpm/min | 103 [90, 119] | 97 [88, 110] | 0.24 |
| Heart rate-low, bpm/min | 67 [58, 78] | 70 [62, 80] | 0.16 |
| systolic blood pressure- max, mmHg | 150 [134, 169] | 140 [127, 154] | 0.44 |
| systolic blood pressure - min, mmHg | 90 [78, 104] | 85 [76, 95] | 0.3 |
| mean blood pressure - max, mmHg | 101 [90, 113] | 93 [85, 103] | 0.49 |
| mean blood pressure - min, mmHg | 61 [53, 70] | 58 [53, 64] | 0.29 |
| diastole blood pressure - max, mmHg | 76 [66, 88] | 70 [61, 80] | 0.39 |
| diastole blood pressure - min, mmHg | 47 [40, 55] | 45 [40, 51] | 0.21 |
| Body temperature - max, ℃ | 37.7 [37.2, 38.3] | 37.9 [37.4, 38.3] | 0.07 |
| Body temperature - min, ℃ | 36.3 [35.9, 36.7] | 36.2 [35.8, 36.6] | 0.06 |
| Respiratory rate - max, /min | 25 [22, 30] | 24 [20, 28] | 0.33 |
| Respiratory rate - min, /min | 12 [10, 15] | 11 [10, 12] | 0.39 |
| GCS.E | 4 [3, 4] | 4 [4, 4] | 0.45 |
| GCS.V | 5 [4, 5] | 5 [5, 5] | 0.49 |
| GCS.M | 6 [6, 6] | 6 [6, 6] | 0.34 |
| Laboratory values |  |  |  |
| Hematocrit - max, % | 33.1 [29.1, 37.6] | 32.8 [30.0, 36.1] | 0.04 |
| Hematocrit - min, % | 30.2 [26.2, 34.9] | 28.8 [26.2, 31.7] | 0.25 |
| WBC - max, 10^9^/l | 10.9 [8.0, 14.7] | 11.3 [8.9, 14.2] | 0.04 |
| WBC – min, 10^9^/l | 9.0 [6.4, 12.2] | 8.7 [6.7, 11.1] | 0.15 |
| Platelet – min, 10^9^/l | 155 [104, 212] | 102 [77, 134] | 0.7 |
| Creatinine - max, mg/dl | 0.9 [0.7, 1.5] | 1.1 [0.8, 1.6] | <0.01 |
| Creatinine - min, mg/dl | 0.8 [0.6, 1.3] | 0.9 [0.7, 1.2] | 0.04 |
| BUN - max, mg/dl | 20.0 [14.0, 32.0] | 19.8 [15.1, 26.7] | 0.23 |
| Na - max, mEq/l | 141 [138, 143] | 143 [140, 146] | 0.46 |
| Na - min, mEq/l | 136 [133, 139] | 138 [135, 140] | 0.32 |
| K - max, mEq/l | 4.3 [4.0, 4.7] | 4.6 [4.3, 4.9] | 0.38 |
| K - min, mEq/l | 3.7 [3.4, 4.0] | 3.8 [3.5, 4.1] | 0.19 |
| Albumin - max, g/dl | 2.9 [2.4, 3.4] | 3.2 [2.9, 3.6] | 0.52 |
| Albumin - min, g/dl | 2.7 [2.2, 3.2] | 2.9 [2.5, 3.3] | 0.31 |
| Bilirubin - max, mg/dl | 0.9 [0.6, 1.3] | 1.2 [0.8, 1.9] | 0.16 |
| Blood glucose - max, mg/dl | 169 [139, 212] | 193 [168, 224] | 0.24 |
| Blood glucose - min, mg/dl | 115 [97, 135] | 125 [108, 141] | 0.22 |
| Lactate, mmol/l | 2.0 [1.3, 3.3] | 2.9 [1.9, 4.4] | 0.25 |

PAC: Pulmonary artery catheter, SMD: Standardized mean difference, GCS: Glasgow coma scale, WBC: White blood cell, BUN: Blood urea nitrogen, Na: sodium, K: potassium

Supplementary Table 3 Vital signs and blood tests in all matched patients

|  | PAC(-) | PAC(+) | SMD |
| --- | --- | --- | --- |
| Number of patients | 5675 | 5675 |  |
| Vital signs |  |  |  |
| Heart rate-max, bpm/min | 99 [88, 112] | 98 [88, 110] | 0.01 |
| Heart rate-low, bpm/min | 68 [59, 78] | 69 [60, 79] | 0.03 |
| systolic blood pressure- max, mmHg | 141 [127, 157] | 142 [128, 157] | 0.01 |
| systolic blood pressure - min, mmHg | 86 [76, 97] | 87 [77, 96] | <0.01 |
| mean blood pressure - max, mmHg | 95 [86, 106] | 95 [87, 105] | 0.01 |
| mean blood pressure - min, mmHg | 59 [52, 65] | 59 [53, 65] | 0.02 |
| diastole blood pressure - max, mmHg | 72 [63, 81] | 71 [62, 81] | 0.01 |
| diastole blood pressure - min, mmHg | 45 [39, 51] | 46 [40, 52] | 0.03 |
| Body temperature - max, ℃ | 37.6 [37.2, 38.1] | 37.8 [37.3, 38.2] | 0.04 |
| Body temperature - min, ℃ | 36.1 [35.7, 36.5] | 36.2 [35.8, 36.6] | 0.04 |
| Respiratory rate - max, /min | 25 [21, 29] | 24 [21, 28] | 0.03 |
| Respiratory rate - min, /min | 11 [10, 13] | 12 [10, 13] | 0.01 |
| GCS.E | 4 [4, 4] | 4 [4, 4] | <0.01 |
| GCS.V | 5 [5, 5] | 5 [5, 5] | <0.01 |
| GCS.M | 6 [6, 6] | 6 [6, 6] | 0.01 |
| Laboratory values |  |  |  |
| Hematocrit - max, % | 33.4 [30.2, 37.2] | 33.5 [30.3, 37.2] | 0.01 |
| Hematocrit - min, % | 29.4 [26.3, 33.0] | 29.3 [26.5, 32.6] | 0.01 |
| WBC - max, 10^9^/l | 11.3 [8.7, 14.5] | 11.5 [9.0, 14.5] | 0.01 |
| WBC - min, 10^9^/l | 8.6 [6.5, 11.3] | 8.8 [6.8, 11.4] | <0.01 |
| Platelet - min, 10^9^/l | 111 [80, 153] | 111 [82, 150] | <0.01 |
| Creatinine - max, mg/dl | 1.1 [0.8, 1.6] | 1.1 [0.8, 1.5] | <0.01 |
| Creatinine - min, mg/dl | 0.9 [0.7, 1.3] | 0.9 [0.7, 1.3] | <0.01 |
| BUN - max, mg/dl | 20.0 [15.0, 27.9] | 20.0 [15.0, 27.4] | <0.01 |
| Na - max, mEq/l | 142 [140, 145] | 142 [140, 145] | 0.02 |
| Na - min, mEq/l | 137 [134, 140] | 137 [134, 140] | <0.01 |
| K - max, mEq/l | 4.5 [4.2, 4.9] | 4.6 [4.3, 4.9] | 0.01 |
| K - min, mEq/l | 3.8 [3.5, 4.1] | 3.8 [3.5, 4.0] | 0.01 |
| Albumin - max, g/dl | 3.2 [2.8, 3.5] | 3.2 [2.8, 3.5] | 0.04 |
| Albumin - min, g/dl | 2.9 [2.5, 3.2] | 2.9 [2.5, 3.2] | 0.03 |
| Bilirubin - max, mg/dl | 1.1 [0.7, 1.7] | 1.1 [0.8, 1.7] | 0.01 |
| Blood glucose - max, mg/dl | 192 [161, 229] | 190 [164, 223] | 0.01 |
| Blood glucose - min, mg/dl | 121 [104, 138] | 122 [105, 139] | 0.01 |
| Lactate, mmol/l | 2.6 [1.6, 4.5] | 2.8 [1.8, 4.5] | 0.02 |

PAC: Pulmonary artery catheter, SMD: Standardized mean difference, GCS: Glasgow coma scale, WBC: White blood cell, BUN: Blood urea nitrogen, Na: sodium, K: potassium

Supplementary Table 4 Patients characteristics in unmatched and matched patients with cardiac disease

|  | | Unmatched | |  | |  | | Matched | |  | |  | |
| --- | --- | --- | --- | --- | --- | --- | --- | --- | --- | --- | --- | --- | --- |
|  | PAC(-) | | PAC(+) | | SMD | | PAC(-) | | PAC(+) | | SMD | |  |
| Number of patients | 11965 | | 11035 | |  | | 4954 | | 4954 | |  | |  |
| Age, median year, IQR | 72 [63, 80] | | 71 [63, 77] | | 0.1 | | 71 [62, 78] | | 71 [63, 78] | | 0.01 | |  |
| Male (%) | 7854 (65.6) | | 7247 (65.7) | | <0.01 | | 3219 (65.0) | | 3227 (65.1) | | <0.01 | |  |
| Height, median cm, IQR | 162 [154, 168] | | 162 [154, 169] | | 0.05 | | 162 [154, 169] | | 162 [154, 169] | | 0.02 | |  |
| Body weight, median kg, IQR | 60.0 [51.0, 70.0] | | 60.0 [51.8, 69.0] | | <0.01 | | 60.3 [51.8, 70.0] | | 60.0 [52.0, 69.3] | | 0.02 | |  |
| BMI, median kg/m^2^, IQR | 23.1 [20.5, 25.8] | | 23.0 [20.6, 25.6] | | 0.03 | | 23.1 [20.8, 25.8] | | 23.1 [20.7, 25.6] | | 0.04 | |  |
| Hospital type (%) |  | |  | | 0.53 | |  | |  | | 0.02 | |  |
| University hospital | 4624 (38.6) | | 7080 (64.2) | |  | | 2523 (50.9) | | 2566 (51.8) | |  | |  |
| Public hospital | 4325 (36.1) | | 2505 (22.7) | |  | | 1643 (33.2) | | 1617 (32.6) | |  | |  |
| Private hospital | 3016 (25.2) | | 1450 (13.1) | |  | | 788 (15.9) | | 771 (15.6) | |  | |  |
| Reason for ICU admission (%) |  | |  | | 1.14 | |  | |  | | 0.05 | |  |
| Open heart surgery |  | |  | |  | |  | |  | |  | |  |
| TAA/AAD | 1781 (14.9) | | 1920 (17.4) | |  | | 1103 (22.3) | | 1062 (21.4) | |  | |  |
| Valve surgery | 1509 (12.6) | | 4165 (37.7) | |  | | 1297 (26.2) | | 1297 (26.2) | |  | |  |
| CABG | 972 ( 8.1) | | 2336 (21.2) | |  | | 862 (17.4) | | 851 (17.2) | |  | |  |
| Valve + CABG | 185 ( 1.5) | | 617 ( 5.6) | |  | | 155 ( 3.1) | | 162 ( 3.3) | |  | |  |
| Other open heart surgery | 1032 ( 8.6) | | 531 ( 4.8) | |  | | 348 ( 7.0) | | 347 ( 7.0) | |  | |  |
| Other cardiac surgery | 340 ( 2.8) | | 213 ( 1.9) | |  | | 164 ( 3.3) | | 149 ( 3.0) | |  | |  |
| Cardiovascular disease |  | |  | |  | |  | |  | |  | |  |
| Cardiogenic shock | 165 ( 1.4) | | 144 ( 1.3) | |  | | 94 ( 1.9) | | 93 ( 1.9) | |  | |  |
| Cardiac arrest | 1627 (13.6) | | 275 ( 2.5) | |  | | 250 ( 5.0) | | 245 ( 4.9) | |  | |  |
| AMI | 1585 (13.2) | | 359 ( 3.3) | |  | | 302 ( 6.1) | | 316 ( 6.4) | |  | |  |
| Aortic aneurysm | 1119 ( 9.4) | | 144 ( 1.3) | |  | | 114 ( 2.3) | | 138 ( 2.8) | |  | |  |
| Congestive heart failure | 1650 (13.8) | | 331 ( 3.0) | |  | | 265 ( 5.3) | | 294 ( 5.9) | |  | |  |
| Root of ICU admission (%) |  | |  | | 0.97 | |  | |  | | 0.04 | |  |
| Transfer from ward | 411 ( 3.4) | | 146 ( 1.3) | |  | | 109 ( 2.2) | | 108 ( 2.2) | |  | |  |
| Transfer from ER | 4223 (35.3) | | 828 ( 7.5) | |  | | 677 (13.7) | | 731 (14.8) | |  | |  |
| After elective surgery | 4243 (35.5) | | 8258 (74.8) | |  | | 3001 (60.6) | | 2997 (60.5) | |  | |  |
| After emergency surgery | 1135 ( 9.5) | | 1135 (10.3) | |  | | 671 (13.5) | | 634 (12.8) | |  | |  |
| Other | 1953 (16.3) | | 668 ( 6.1) | |  | | 496 (10.0) | | 484 ( 9.8) | |  | |  |
| ICU readmission (%) | 618 ( 5.2) | | 276 ( 2.5) | | 0.14 | | 182 ( 3.7) | | 169 ( 3.4) | | 0.01 | |  |
| Comorbidities (%) |  | |  | |  | |  | |  | |  | |  |
| Heart failure | 357 ( 3.0) | | 582 ( 5.3) | | 0.12 | | 164 ( 3.3) | | 192 ( 3.9) | | 0.03 | |  |
| Respiratory failure | 123 ( 1.0) | | 71 ( 0.6) | | 0.04 | | 27 ( 0.5) | | 28 ( 0.6) | | <0.01 | |  |
| Immunosuppression | 275 ( 2.3) | | 152 ( 1.4) | | 0.07 | | 90 ( 1.8) | | 80 ( 1.6) | | 0.02 | |  |
| Hemodialysis | 849 ( 7.1) | | 958 ( 8.7) | | 0.06 | | 362 ( 7.3) | | 373 ( 7.5) | | 0.01 | |  |
| APACHE III median, IQR | 61 [48, 80] | | 61 [50, 73] | | 0.19 | | 61 [49, 76] | | 61 [49, 74] | | 0.03 | |  |
| Catecholamine use (%) |  | |  | |  | |  | |  | |  | |  |
| Dopamine | 1716 (14.3) | | 2822 (25.6) | | 0.28 | | 1089 (22.0) | | 1098 (22.2) | | <0.01 | |  |
| Noradrenaline | 3450 (28.8) | | 5169 (46.8) | | 0.38 | | 1881 (38.0) | | 1869 (37.7) | | <0.01 | |  |
| Dobutamine | 3257 (27.2) | | 6976 (63.2) | | 0.78 | | 2409 (48.6) | | 2387 (48.2) | | 0.01 | |  |
| Adrenaline | 265 ( 2.2) | | 343 ( 3.1) | | 0.06 | | 135 ( 2.7) | | 121 ( 2.4) | | 0.02 | |  |

PAC: Pulmonary artery catheter, SMD: Standardized mean difference, IQR: Interquartile range, TAA: Thoracic aortic aneurysm, AAD: Acute Aortic dissection, CABG: Coronary artery bypass grafting, AMI: Acute myocardial infarction, BMI: Body mass index, ICU: Intensive care unit, ER: Emergency room, SMD: Standardized mean difference, APACHE: Acute physiologic assessment and chronic health evaluation, Catecholamine use: within 24 hours after ICU admission.

Supplementary Table 5 Interventions and outcome in unmatched and matched patients with cardiac disease

|  | Unmatched | |  | |  | | Matched | | |  |
| --- | --- | --- | --- | --- | --- | --- | --- | --- | --- | --- |
|  | PAC(-) | PAC(+) | | P value | | PAC(-) | | PAC(+) | P value | |
| Number of patients | 11965 | 11035 | |  | | 4954 | | 4954 |  | |
| HFNC (%) | 1727 (14.4) | 2208 (20.0) | | <0.01 | | 941 (19.0) | | 996 (20.1) | 0.17 | |
| NPPV (%) | 1931 (16.1) | 1778 (16.1) | | 0.97 | | 621 (12.5) | | 796 (16.1) | <0.01 | |
| Mechanical ventilation (%) | 6666 (55.7) | 10353 (93.8) | | <0.01 | | 3861 (77.9) | | 4383 (88.5) | <0.01 | |
| Duration, median hours, IQR | 26 [13, 89] | 17 [9, 41] | | <0.01 | | 17 [9, 57] | | 17 [8, 55] | 0.01 | |
| VV ECMO (%) | 58 ( 0.5) | 41 ( 0.4) | | 0.23 | | 33 ( 0.7) | | 21 ( 0.4) | 0.13 | |
| IABP, % | 955 ( 8.0) | 1215 (11.0) | | <0.01 | | 434 ( 8.8) | | 684 (13.8) | <0.01 | |
| VA ECMO (%) | 334 ( 2.8) | 477 ( 4.3) | | <0.01 | | 157 ( 3.2) | | 303 ( 6.1) | <0.01 | |
| IRRT (%) | 898 ( 7.5) | 925 ( 8.4) | | 0.01 | | 350 ( 7.1) | | 370 ( 7.5) | 0.46 | |
| CRRT (%) | 1164 ( 9.7) | 1271 (11.5) | | <0.01 | | 489 ( 9.9) | | 578 (11.7) | <0.01 | |
| ICU mortality (%) | 1467 (12.3) | 580 ( 5.3) | | <0.01 | | 383 ( 7.7) | | 337 ( 6.8) | 0.08 | |
| Hospital mortality (%) | 596 ( 5.0) | 304 ( 2.8) | | <0.01 | | 183 ( 3.7) | | 168 ( 3.4) | 0.45 | |
| ICU length of stay, median days, IQR | 21 [13, 35] | 25 [18, 40] | | <0.01 | | 22 [15, 35] | | 25 [17, 39] | <0.01 | |
| Hospital length of stay, median days, IQR | 3 [1, 5] | 3 [2, 5] | | <0.01 | | 3 [2, 5] | | 3 [2, 6] | 0.02 | |

PAC: Pulmonary artery catheter, IQR: Interquartile range, HFNC: High flow nasal cannula, NPPV: Non-invasive positive pressure ventilation, IABP: Intra-aortic balloon pump, VA ECMO: Venoarterial extracorporeal membrane oxygenation, VV ECMO: Venovenous extracorporeal membrane oxygenation, IRRT: Intermittent renal replacement therapy, CRRT: Continuous renal replacement therapy, ICU: Intensive care unit

Supplementary Table 6 Patients characteristics in unmatched and matched patients with post open heart surgery

|  | | Unmatched | |  | |  | Matched | | |  |  | |  |
| --- | --- | --- | --- | --- | --- | --- | --- | --- | --- | --- | --- | --- | --- |
|  | PAC(-) | | PAC(+) | | SMD | | | PAC(-) | PAC(+) | | | SMD | |
| Number of patients | 5479 | | 9569 | |  | | | 3665 | 3665 | | |  | |
| Age, median year, IQR | 72 [63, 79] | | 71 [63, 77] | | 0.05 | | | 71 [62, 78] | 71 [63, 77] | | | 0.02 | |
| Male (%) | 3521 (64.3) | | 6269 (65.5) | | 0.03 | | | 2392 (65.3) | 2393 (65.3) | | | <0.01 | |
| Height, median cm, IQR | 162 [154, 168] | | 162 [154, 168] | | 0.06 | | | 162 [154, 169] | 162 [154, 169] | | | 0.04 | |
| Body weight, median kg, IQR | 60.0 [51.4, 69.6] | | 60.0 [51.7, 68.5] | | 0.02 | | | 60.6 [52.1, 70.0] | 60.0 [51.8, 68.8] | | | 0.06 | |
| BMI, median kg/m^2^, IQR | 23.2 [20.8, 25.8] | | 23.0 [20.6, 25.5] | | 0.06 | | | 23.3 [20.9, 25.9] | 22.8 [20.5, 25.5] | | | 0.09 | |
| Hospital type (%) |  | |  | | 0.46 | | |  |  | | | 0.03 | |
| University hospital | 2442 (44.6) | | 6380 (66.7) | |  | | | 1909 (52.1) | 1887 (51.5) | | |  | |
| Public hospital | 2001 (36.5) | | 2049 (21.4) | |  | | | 1223 (33.4) | 1265 (34.5) | | |  | |
| Private hospital | 1036 (18.9) | | 1140 (11.9) | |  | | | 533 (14.5) | 513 (14.0) | | |  | |
| Reason for ICU admission (%) |  | |  | | 0.59 | | |  |  | | | 0.02 | |
| Open heart surgery |  | |  | |  | | |  |  | | |  | |
| TAA/AAD | 1781 (32.5) | | 1920 (20.1) | |  | | | 1076 (29.4) | 1099 (30.0) | | |  | |
| Valve surgery | 1509 (27.5) | | 4165 (43.5) | |  | | | 1255 (34.2) | 1231 (33.6) | | |  | |
| CABG | 972 (17.7) | | 2336 (24.4) | |  | | | 835 (22.8) | 825 (22.5) | | |  | |
| Valve + CABG | 185 ( 3.4) | | 617 ( 6.4) | |  | | | 146 ( 4.0) | 152 ( 4.1) | | |  | |
| Other open heart surgery | 1032 (18.8) | | 531 ( 5.5) | |  | | | 353 ( 9.6) | 358 ( 9.8) | | |  | |
| Root of ICU admission (%) |  | |  | | 0.24 | | |  |  | | | 0.01 | |
| After elective surgery | 4230 (77.2) | | 8257 (86.3) | |  | | | 2936 (80.1) | 2943 (80.3) | | |  | |
| After emergency surgery | 1013 (18.5) | | 1087 (11.4) | |  | | | 592 (16.2) | 589 (16.1) | | |  | |
| Other | 236 ( 4.3) | | 225 ( 2.4) | |  | | | 137 ( 3.7) | 133 ( 3.6) | | |  | |
| ICU readmission (%) | 244 ( 4.5) | | 211 ( 2.2) | | 0.13 | | | 117 ( 3.2) | 116 ( 3.2) | | | <0.01 | |
| Comorbidities (%) |  | |  | |  | | |  |  | | |  | |
| Heart failure | 120 ( 2.2) | | 455 ( 4.8) | | 0.14 | | | 83 ( 2.3) | 90 ( 2.5) | | | 0.01 | |
| Respiratory failure | 13 ( 0.2) | | 52 ( 0.5) | | 0.05 | | | 11 ( 0.3) | 9 ( 0.2) | | | 0.01 | |
| Immunosuppression | 65 ( 1.2) | | 119 ( 1.2) | | 0.01 | | | 47 ( 1.3) | 47 ( 1.3) | | | <0.01 | |
| Hemodialysis | 329 ( 6.0) | | 873 ( 9.1) | | 0.12 | | | 249 ( 6.8) | 257 ( 7.0) | | | 0.01 | |
| APACHE III median, IQR | 57 [47, 71] | | 60 [50, 71] | | 0.08 | | | 58 [48, 71] | 59 [48, 70] | | | 0.01 | |
| Catecholamine use (%) |  | |  | |  | | |  |  | | |  | |
| Dopamine | 1148 (21.0) | | 2564 (26.8) | | 0.14 | | | 912 (24.9) | 882 (24.1) | | | 0.02 | |
| Noradrenaline | 1589 (29.0) | | 4456 (46.6) | | 0.37 | | | 1305 (35.6) | 1313 (35.8) | | | <0.01 | |
| Dobutamine | 2183 (39.8) | | 6104 (63.8) | | 0.49 | | | 1833 (50.0) | 1815 (49.5) | | | 0.01 | |
| Adrenaline | 95 ( 1.7) | | 306 ( 3.2) | | 0.09 | | | 78 ( 2.1) | 82 ( 2.2) | | | 0.01 | |

PAC: Pulmonary artery catheter, SMD: Standardized mean difference, IQR: Interquartile range, TAA: Thoracic aortic aneurysm, AAD: Acute Aortic dissection, CABG: Coronary artery bypass grafting, AMI: Acute myocardial infarction, BMI: Body mass index, ICU: Intensive care unit, ER: Emergency room, SMD: Standardized mean difference, APACHE: Acute physiologic assessment and chronic health evaluation, Catecholamine use: within 24 hours after ICU admission.

Supplementary Table 7 Interventions and outcome in unmatched and matched patients with post open heart surgery

|  | Unmatched | |  | |  | Matched | | |  |
| --- | --- | --- | --- | --- | --- | --- | --- | --- | --- |
|  | PAC(-) | PAC(+) | | P value | PAC(-) | | PAC(+) | P value | |
| Number of patients | 5479 | 9569 | |  | 3665 | | 3665 |  | |
| HFNC (%) | 1057 (19.3) | 1929 (20.2) | | 0.21 | 764 (20.8) | | 750 (20.5) | 0.71 | |
| NPPV (%) | 523 ( 9.5) | 1436 (15.0) | | <0.01 | 368 (10.0) | | 500 (13.6) | <0.01 | |
| Mechanical ventilation (%) | 4119 (75.2) | 9374 (98.0) | | <0.01 | 3139 (85.6) | | 3529 (96.3) | <0.01 | |
| Duration, median hours, IQR | 15 [7, 37] | 16 [7, 27] | | 0.69 | 15 [7, 34] | | 15 [6, 24] | <0.01 | |
| VV ECMO (%) | 25 ( 0.5) | 25 ( 0.3) | | 0.06 | 19 ( 0.5) | | 11 ( 0.3) | 0.2 | |
| IABP (%) | 291 ( 5.3) | 675 ( 7.1) | | <0.01 | 223 ( 6.1) | | 247 ( 6.7) | 0.27 | |
| VA ECMO (%) | 84 ( 1.5) | 183 ( 1.9) | | 0.1 | 60 ( 1.6) | | 84 ( 2.3) | 0.05 | |
| IRRT (%) | 331 ( 6.0) | 795 ( 8.3) | | <0.01 | 238 ( 6.5) | | 235 ( 6.4) | 0.92 | |
| CRRT (%) | 386 ( 7.0) | 954 (10.0) | | <0.01 | 289 ( 7.9) | | 324 ( 8.8) | 0.15 | |
| ICU mortality (%) | 197 ( 3.6) | 320 ( 3.3) | | 0.44 | 119 ( 3.2) | | 138 ( 3.8) | 0.25 | |
| Hospital mortality (%) | 99 ( 1.8) | 157 ( 1.6) | | 0.49 | 64 ( 1.7) | | 62 ( 1.7) | 0.93 | |
| ICU length of stay, median days, IQR | 21 [15, 33] | 25 [19, 39] | | <0.01 | 22 [16, 33] | | 24 [17, 38] | <0.01 | |
| Hospital length of stay, median days, IQR | 3 [1, 4] | 3 [1, 5] | | <0.01 | 3 [2, 5] | | 3 [1, 5] | 0.14 | |

PAC: Pulmonary artery catheter, IQR: Interquartile range, HFNC: High flow nasal cannula, NPPV: Non-invasive positive pressure ventilation, IABP: Intra-aortic balloon pump, VA ECMO: Venoarterial extracorporeal membrane oxygenation, VV ECMO: Venovenous extracorporeal membrane oxygenation, IRRT: Intermittent renal replacement therapy, CRRT: Continuous renal replacement therapy, ICU: Intensive care unit

Supplementary Table 8 Patients characteristics in unmatched and matched patients with cardiac disease with shock and/or device

|  | | Unmatched | |  | |  | | Matched | |  | |  |
| --- | --- | --- | --- | --- | --- | --- | --- | --- | --- | --- | --- | --- |
|  | PAC(-) | | PAC(+) | | SMD | | PAC(-) | | PAC(+) | | SMD | |
| Number of patients | 6155 | | 9209 | |  | | 3721 | | 3721 | |  | |
| Age, median year, IQR | 72 [63, 79] | | 71 [63, 77] | | 0.07 | | 71 [63, 78] | | 71 [62, 78] | | 0.02 | |
| Male (%) | 4099 (66.6) | | 6145 (66.7) | | <0.01 | | 2485 (66.8) | | 2474 (66.5) | | 0.01 | |
| Height, median cm, IQR | 162 [154, 168] | | 162 [155, 169] | | 0.07 | | 162 [155, 169] | | 162 [154, 169] | | 0.02 | |
| Body weight, median kg, IQR | 59.7 [50.8, 69.2] | | 60.3 [52.0, 69.0] | | 0.04 | | 60.0 [51.7, 69.9] | | 60.2 [52.0, 69.4] | | 0.01 | |
| BMI, median kg/m^2^, IQR | 22.9 [20.4, 25.7] | | 23.0 [20.6, 25.6] | | <0.01 | | 23.1 [20.6, 25.7] | | 23.1 [20.7, 25.7] | | 0.01 | |
| Hospital type (%) |  | |  | | 0.56 | |  | |  | | 0.04 | |
| University hospital | 2534 (41.2) | | 6145 (66.7) | |  | | 1891 (50.8) | | 1893 (50.9) | |  | |
| Public hospital | 2441 (39.7) | | 1703 (18.5) | |  | | 1183 (31.8) | | 1225 (32.9) | |  | |
| Private hospital | 1180 (19.2) | | 1361 (14.8) | |  | | 647 (17.4) | | 603 (16.2) | |  | |
| Reason for ICU admission (%) |  | |  | | 0.87 | |  | |  | | 0.05 | |
| Open heart surgery |  | |  | |  | |  | |  | |  | |
| TAA/AAD | 842 (13.7) | | 1513 (16.4) | |  | | 681 (18.3) | | 734 (19.7) | |  | |
| Valve surgery | 1134 (18.4) | | 3506 (38.1) | |  | | 1003 (27.0) | | 968 (26.0) | |  | |
| CABG | 812 (13.2) | | 1982 (21.5) | |  | | 707 (19.0) | | 698 (18.8) | |  | |
| Valve + CABG | 144 ( 2.3) | | 554 ( 6.0) | |  | | 123 ( 3.3) | | 124 ( 3.3) | |  | |
| Other open heart surgery | 419 ( 6.8) | | 452 ( 4.9) | |  | | 257 ( 6.9) | | 268 ( 7.2) | |  | |
| Other cardiac surgery | 161 ( 2.6) | | 176 ( 1.9) | |  | | 114 ( 3.1) | | 111 ( 3.0) | |  | |
| Cardiovascular disease |  | |  | |  | |  | |  | |  | |
| Cardiogenic shock | 146 ( 2.4) | | 143 ( 1.6) | |  | | 90 ( 2.4) | | 97 ( 2.6) | |  | |
| Cardiac arrest | 1170 (19.0) | | 254 ( 2.8) | |  | | 253 ( 6.8) | | 228 ( 6.1) | |  | |
| AMI | 600 ( 9.7) | | 248 ( 2.7) | |  | | 211 ( 5.7) | | 216 ( 5.8) | |  | |
| Aortic aneurysm | 84 ( 1.4) | | 124 ( 1.3) | |  | | 55 ( 1.5) | | 55 ( 1.5) | |  | |
| Congestive heart failure | 643 (10.4) | | 257 ( 2.8) | |  | | 227 ( 6.1) | | 222 ( 6.0) | |  | |
| Root of ICU admission (%) |  | |  | | 0.78 | |  | |  | | 0.03 | |
| Transfer from ward | 203 ( 3.3) | | 128 ( 1.4) | |  | | 85 ( 2.3) | | 86 ( 2.3) | |  | |
| Transfer from ER | 1559 (25.3) | | 642 ( 7.0) | |  | | 509 (13.7) | | 514 (13.8) | |  | |
| After elective surgery | 2593 (42.1) | | 6879 (74.7) | |  | | 2214 (59.5) | | 2197 (59.0) | |  | |
| After emergency surgery | 643 (10.4) | | 972 (10.6) | |  | | 477 (12.8) | | 506 (13.6) | |  | |
| Other | 1157 (18.8) | | 588 ( 6.4) | |  | | 436 (11.7) | | 418 (11.2) | |  | |
| ICU readmission (%) | 365 ( 5.9) | | 246 ( 2.7) | | 0.16 | | 158 ( 4.2) | | 150 ( 4.0) | | 0.01 | |
| Comorbidities (%) |  | |  | |  | |  | |  | |  | |
| Heart failure | 210 ( 3.4) | | 549 ( 6.0) | | 0.12 | | 153 ( 4.1) | | 163 ( 4.4) | | 0.01 | |
| Respiratory failure | 73 ( 1.2) | | 67 ( 0.7) | | 0.05 | | 31 ( 0.8) | | 28 ( 0.8) | | 0.01 | |
| Immunosuppression | 158 ( 2.6) | | 127 ( 1.4) | | 0.09 | | 61 ( 1.6) | | 64 ( 1.7) | | 0.01 | |
| Hemodialysis | 494 ( 8.0) | | 814 ( 8.8) | | 0.03 | | 282 ( 7.6) | | 279 ( 7.5) | | <0.01 | |
| APACHE III median, IQR | 69 [53, 94] | | 61 [51, 74] | | 0.45 | | 64 [51, 79] | | 63 [51, 78] | | 0.04 | |
| Catecholamine use (%) |  | |  | |  | |  | |  | |  | |
| Dopamine | 1716 (27.9) | | 2822 (30.6) | | 0.06 | | 1133 (30.4) | | 1128 (30.3) | | <0.01 | |
| Noradrenaline | 3450 (56.1) | | 5169 (56.1) | | <0.01 | | 1921 (51.6) | | 1952 (52.5) | | 0.02 | |
| Dobutamine | 3257 (52.9) | | 6976 (75.8) | | 0.49 | | 2415 (64.9) | | 2393 (64.3) | | 0.01 | |
| Adrenaline | 265 ( 4.3) | | 343 ( 3.7) | | 0.03 | | 124 ( 3.3) | | 123 ( 3.3) | | <0.01 | |

PAC: Pulmonary artery catheter, SMD: Standardized mean difference, IQR: Interquartile range, TAA: Thoracic aortic aneurysm, AAD: Acute Aortic dissection, CABG: Coronary artery bypass grafting, AMI: Acute myocardial infarction, BMI: Body mass index, ICU: Intensive care unit, ER: Emergency room, SMD: Standardized mean difference, APACHE: Acute physiologic assessment and chronic health evaluation, Catecholamine use: within 24 hours after ICU admission.

Supplementary Table 9 Interventions and outcome in unmatched and matched patients with cardiac disease with shock and/or device

|  | Unmatched | | |  | |  | Matched | | |  |
| --- | --- | --- | --- | --- | --- | --- | --- | --- | --- | --- |
|  | PAC(-) | PAC(+) | P value | | PAC(-) | | | PAC(+) | P value | |
| Number of patients | 6155 | 9209 |  | | 3721 | | | 3721 |  | |
| HFNC (%) | 989 (16.1) | 1870 (20.3) | <0.01 | | 717 (19.3) | | | 740 (19.9) | 0.52 | |
| NPPV (%) | 952 (15.5) | 1561 (17.0) | 0.02 | | 527 (14.2) | | | 642 (17.3) | <0.01 | |
| Mechanical ventilation (%) | 4802 (78.0) | 8798 (95.5) | <0.01 | | 3110 (83.6) | | | 3389 (91.1) | <0.01 | |
| Duration, median hours, IQR | 28 [14, 96] | 17 [11, 44] | <0.01 | | 18 [11, 62] | | | 18 [10, 66] | 0.79 | |
| VV ECMO (%) | 58 ( 0.9) | 41 ( 0.4) | <0.01 | | 31 ( 0.8) | | | 21 ( 0.6) | 0.21 | |
| IABP (%) | 955 (15.5) | 1215 (13.2) | <0.01 | | 458 (12.3) | | | 711 (19.1) | <0.01 | |
| VA ECMO (%) | 334 ( 5.4) | 477 ( 5.2) | 0.53 | | 149 ( 4.0) | | | 311 ( 8.4) | <0.01 | |
| IRRT (%) | 541 ( 8.8) | 817 ( 8.9) | 0.88 | | 300 ( 8.1) | | | 301 ( 8.1) | 1 | |
| CRRT (%) | 839 (13.6) | 1166 (12.7) | 0.08 | | 443 (11.9) | | | 504 (13.5) | 0.04 | |
| ICU mortality (%) | 1069 (17.4) | 548 ( 6.0) | <0.01 | | 367 ( 9.9) | | | 332 ( 8.9) | 0.18 | |
| Hospital mortality (%) | 495 ( 8.0) | 294 ( 3.2) | <0.01 | | 181 ( 4.9) | | | 177 ( 4.8) | 0.87 | |
| ICU length of stay, median days, IQR | 23 [16, 41] | 26 [19, 42] | <0.01 | | 23 [16, 38] | | | 26 [18, 42] | <0.01 | |
| Hospital length of stay, median days, IQR | 3 [2, 6] | 3 [2, 5] | <0.01 | | 3 [2, 6] | | | 3 [2, 6] | 0.08 | |

PAC: Pulmonary artery catheter, IQR: Interquartile range, HFNC: High flow nasal cannula, NPPV: Non-invasive positive pressure ventilation, IABP: Intra-aortic balloon pump, VA ECMO: Venoarterial extracorporeal membrane oxygenation, VV ECMO: Venovenous extracorporeal membrane oxygenation, IRRT: Intermittent renal replacement therapy, CRRT: Continuous renal replacement therapy, ICU: Intensive care unit
